# Risk Factors for Alcohol Consumption after Starting Assisted Reproductive Technology Treatment among Japanese Women: Japan-Female Employment and Mental Health in Assisted Reproductive Technology (J-FEMA) Study

**DOI:** 10.3390/ijerph20247152

**Published:** 2023-12-07

**Authors:** Mamiko Sato, Motoki Endo, Kiyohide Tomooka, Keiji Kuroda, Yuito Ueda, Setsuko Sato, Yuko Ikemoto, Yuya Imai, Kiyomi Mitsui, Atsushi Tanaka, Rikikazu Sugiyama, Koji Nakagawa, Yuichi Sato, Yasushi Kuribayashi, Mari Kitade, Atsuo Itakura, Satoru Takeda, Takeshi Tanigawa

**Affiliations:** 1 Department of Public Health, Juntendo University Graduate School of Medicine, Tokyo 113-0033, Japan; 2 Department of Obstetrics and Gynaecology, Juntendo University Faculty of Medicine, Tokyo 113-8421, Japan; 3 Centre for Reproductive Medicine and Endoscopy, Sugiyama Clinic Marunouchi, Tokyo 100-0065, Japan; 4 Department of Hygiene, Public Health, and Preventive Medicine, Showa University, Tokyo 142-8555, Japan; 5 Saint Mother Hospital Infertility Clinic, Fukuoka 807-0825, Japan; 6 Centre for Reproductive Medicine and Implantation Research, Sugiyama Clinic Shinjuku, Tokyo 116-0023, Japan; 7 Takasaki ART Clinic, Gunma 370-0831, Japan

**Keywords:** alcohol, assisted reproductive technology, habitual drinking, healthy pregnancy, fertility, social factors

## Abstract

Aims: To determine the association between drinking habits and social factors among women undergoing assisted reproductive technology (ART) treatment in Japan. Methods: The study participants who provided answers for the questionnaire concerning alcohol consumption were 1017 female patients undergoing ART treatment were enrolled in the Japan-Female Employment and Mental Health in assisted reproductive technology (J-FEMA) study between August and December 2018. Patient characteristics, including demographic, clinical, and socioeconomic status, were assessed using a self-administered questionnaire which was distributed only once during the period, regardless of their first or follow-up examination. We defined current drinkers who drank ≥46 g of ethanol per week as the habitual drinking group. The risk factors for habitual drinking were analyzed using multivariable-adjusted logistic regression. Results: The proportion of habitual drinkers was 15.5% in this study population. The multivariable-adjusted odds ratios (95% confidence interval) for habitual drinking were 2.27 (0.99–5.21) for women aged ≥35 years versus those <35 years, 4.26 (1.98–9.16) for women having partners who currently drink compared to those with partners without current drinking, 1.84 (1.08–3.12) for women without a history of childbirth versus those with, and 1.77 (1.00–3.14) for working women compared with those not working. Conclusions: In our study, habitual drinking among women undergoing ART treatment was significantly associated with older age, no history of childbirth, partner’s current drinking status, and working.

## 1. Introduction

Over the past 50 years, there has been a significant decline in the global fertility rate [1]. The fertility rate in Japan has declined over the years and is extremely low by international standards. The total fertility rate in Japan was 1.76 in 1985, 1.42 in 1995, and 1.36 in 2019, while the world total fertility rate was 2.42 in 2018 [1,2]. As women become more educated, they give birth later in Japan [2]. As women’s ovarian function declines with age, and the decline accelerates after the age of 35, the number of women undergoing fertility treatment is increasing [3,4]. According to Japan’s National Assisted Reproductive Technology (ART) Registry Database, ART treatments, including in vitro fertilization (IVF), have more than doubled over the past decade, rising from 161,164 in 2007 to 449,900 in 2020 [5,6].

Infertility can be caused by female or male factors, by a combination of them, by genetic factors, or as a result of idiopathic infertility [7]. Female infertility can be caused by ovarian disorder, oviductal disorder, or uterine disorders [7]. The considerable interest in the impact of preventable factors, such as modifiable lifestyle and behavioral factors, on a woman’s fertility has been previously summarized [8]. It is well known that alcohol consumption and abuse during pregnancy can have detrimental effects on the fetus, including miscarriage, stillbirth, or fetal alcohol spectrum disorders [8,9]. Several studies have investigated the association between alcohol consumption and ART outcomes and have consistently shown that alcohol has a detrimental effect on ART outcomes [10,11,12,13,14,15]. A large prospective study showed women drinking ≥50 g of alcohol a week at the time of IVF cycle start had a significant decline in live birth rate compared with those drinking <50 g of alcohol and reported associations of alcohol consumption with an increased likelihood of failed fertilization and failed implantation [11]. A prospective cohort study on women enrolled in ART demonstrated that alcohol consumption during the year and the week before the IVF was associated with a decrease in the number of oocytes retrieved and an increase in the risk of miscarriage [12]. This study also demonstrated that alcohol consumption during the month preceding the IVF tended to increase the risk of not achieving a pregnancy. In contrast, a cross-sectional study investigating the impact of lifestyle change on ART outcomes demonstrated that women who abstained from drinking or reduced alcohol intake had 2-fold higher odds of becoming pregnant compared to women who maintained their habitual alcohol consumption prior to ART [13]. Additionally, a dose-dependent negative effect of alcohol consumption on embryo quality and reduced odds for blastocyst formation were also detected [14,15]. Thus, given that alcohol consumption is a modifiable risk factor for adverse perinatal events and infertility, government bodies in many countries have issued drinking guidelines regarding alcohol consumption by women who may become pregnant, are pregnant, or are breastfeeding [16].

According to the WHO’s Global status report on alcohol and health 2014 and 2018, the women’s drinking rate, the amount of alcohol consumed by females, and the percentage of female heavy drinkers have increased in Japan and are high compared to the world and the West Pacific Region (WPR) [17,18]. In particular, the percentage of women who had drinking habits (drinking ≥3 days a week and consuming ≥20 g alcohol per drinking day) increased from 3.4% in 1993 to 5.2% in 2018 for women in their 20s, from 7.1% to 8.6% for women in their 30s, and from 9.9% to 14.7% for women in their 40s, despite being childbearing and in childrearing years [19,20].

To our knowledge, there are no epidemiological findings on the drinking habits of Japanese women undergoing ART treatment. Determining the factors associated with alcohol consumption among women undergoing ART treatment may help in developing prevention and intervention strategies to reduce alcohol consumption among women who wish to become pregnant. This study aimed to determine the association between drinking habits and social factors among women undergoing ART treatment in Japan.

## 2. Materials and Methods

### 2.1. Patients

**Figure 1:**
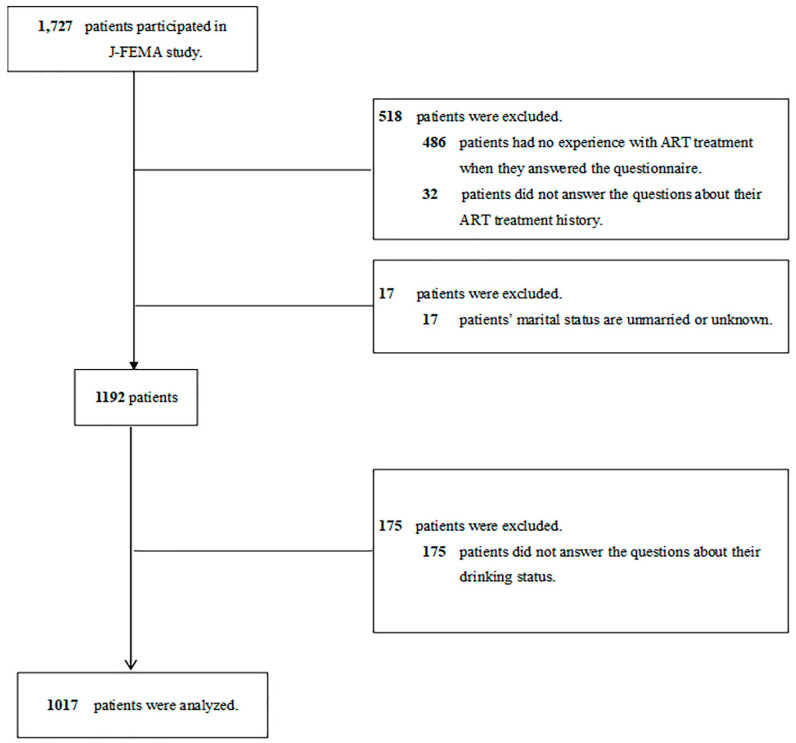
Flow chart of study population included in the analysis.

This cross-sectional study was conducted as part of the Japan-Female Employment and Mental Health in ART (J-FEMA) study. The study participants were 1727 women aged 20 years and over who underwent fertility treatment between August and December 2018 at four fertility clinics: Sugiyama Clinic Shinjuku in Tokyo, Sugiyama Clinic Marunouchi in Tokyo, St. Mother’s Obstetrics and Gynaecology Clinic in Fukuoka, and Takasaki ART Clinic in Gunma (details of the J-FEMA study are shown in a previous report) [21]. Of these patients, we excluded 535 who had no experience with ART treatment (*n* = 486) and did not answer the question about the history of their ART treatment (*n* = 32) and marital status (*n* = 17). We further excluded those who did not answer the questions regarding their drinking history (*n* = 175). Finally, 1017 patients were eligible for analysis (Figure 1). The study protocol was reviewed and approved by the Ethics Committee of Juntendo University (No. 2020100). Written informed consent was obtained from all participants, and no compensation was received for their participation. All procedures involving human participants were performed in accordance with the Declaration of Helsinki.

### 2.2. Questionnaire and Variables

In this study, patients completed an anonymous written self-administered questionnaire. The questionnaire was distributed to the patients on paper and face-to-face at the time of the visit to the clinics. Patients provided responses to the questionnaire only once during the period, regardless of whether it was their first or follow-up examination. The patients were asked to describe their drinking status as never drinker, ex-drinker, and current drinker. We also assessed the usual weekly alcohol intake in units of *go*, a traditional Japanese unit of volume corresponding to approximately 23 g of ethanol. One *go* is 180 mL of sake and corresponds to one bottle (633 mL) of beer, two single shots (75 mL) of whiskey, or two glasses (180 mL) of wine. We defined current drinkers who drank ≥2 *go* (46 g of ethanol) per week as the habitual drinking group.

We also assessed the patients’ age (<35 and ≥35 years), educational background (higher educational background including university/graduate school, and lower educational background including junior high school/high school/junior college/vocational school/others), partner’s drinking status (non-drinking and drinking), duration of infertility (<2 years and ≥2 years), IVF cycle (<6 cycles and ≥6 cycles), history of childbirth (yes and no), psychological distress, insomnia status, employment type at the time of answering the questionnaire (non-worker and worker), and company size at the time of answering the questionnaire (<50, 50–999, and ≥1000 employees).

The questions regarding their partners’ drinking status were described as never drinker, ex-drinker, and current drinker. We defined ‘never drinker’ and ‘ex-drinker’ as the not drinking group and ‘current drinker’ as the drinking group. Psychological distress was assessed using the K6 score, which estimates how frequently respondents have experienced symptoms of psychological distress during the previous 30 days, using a cut-off score of K6 ≥ 13 points as a high probability of severe psychological distress [22]. Insomnia status was assessed by using the Insomnia Severity Index in Japan (ISI-J) score, which consists of seven questions on insomnia symptoms in the past two weeks, using the cut-off score of ISI-J ≥ 10 to define insomniac women [23].

### 2.3. Statistical Analysis

Pearson’s chi-square test was used to describe demographic characteristics according to drinking habits. Potential risk factors for habitual drinking during ART treatment were examined using a multivariable logistic regression model after adjusting for variables that were significant in the crude analysis, including patient age, educational background, partner’s drinking status, history of childbirth, and employment type at the time of answering the questionnaire.

All statistical analyses were performed using IBM SPSS for Windows (version 25.0; IBM Corp., Armonk, NY, USA). All probability values for statistical tests were two-tailed, and values of *p* < 0.05 were considered statistically significant.

## 3. Results

Among 1017 women on ART treatment, the mean (SD) age was 37.5 (4.8) years, and the number (%) of current drinkers and habitual drinkers were 329 (32.4%) and 158 (15.5%). Table 1 describes the demographic characteristics of the study population according to their level of drinking. Patients with habitual drinking were significantly older, had a higher educational background, had partners who drank, did not have a history of childbirth, and were working at the time of the survey, compared to women without habitual drinking.

The multivariable-adjusted odds ratios (ORs) and 95% confidence intervals (CIs) of habitual drinking for potential risk factors are shown in Table 2. The multivariable-adjusted OR (95% CI) of habitual drinking for women aged ≥ 35 years versus those < 35 years was 2.27 (0.99–5.21). The respective OR (95% CI) for women having partners with current drinking versus those having partners without current drinking was 4.26 (1.98–9.16). The respective OR (95% CI) for women who had never experienced childbirth versus those who had experienced childbirth was 1.84 (1.08–3.12). Finally, the respective OR (95% CI) for working versus non-working women was 1.77 (1.00–3.14).

## 4. Discussion

In the present study, the proportion of habitual drinkers among women on ART treatment was 15%, and habitual drinking in this setting was significantly associated with older age, no history of childbirth, partner’s drinking status, and working status. To the best of our knowledge, this is the first epidemiological study to investigate the drinking habits of Japanese women undergoing ART treatment.

In the present study, 32.4% (329/1017) of women on ART treatment consumed any amount of alcohol, and 15.5% (158/1017) consumed two *go* or more of alcohol per week. According to the National Health and Nutrition Survey in Japan, the age-specific proportions of alcohol consumption among Japanese women aged 20–29 years, 30–39 years, and 40–49 years, were 67.5%, 51.7%, and 61.6%, respectively [24]. The prevalence of women on ART treatment who consumed alcohol in our study was lower than that of the general population, suggesting that women on ART treatment tended to moderate their alcohol consumption. In addition, a previous study in the United States showed that the proportion of women on ART treatment who consumed 50 g or more alcohol per week was 22.4% [11]. Although this figure is relatively higher than that reported in our study, we should interpret these results with caution because, considering that Japanese people tend to have lower activity of alcohol-metabolizing enzymes than non-Asian ethnic groups and are smaller in stature than Americans, Japanese are more susceptible to alcohol toxicity than Americans if Japanese women drank as much as American women [25,26]. However, because few studies have reported on the drinking habits of women undergoing fertility treatment, we could not determine whether the figure reported in our study was high or not. Hence, further research is needed on the actual drinking habits of Asian women during infertility treatment, as well as in early pregnancy.

The prevalence of habitual drinking was higher in women aged ≥35 years than in those aged <35 years. This result was similar to previous literature investigating the drinking habits of Japanese women before and after becoming aware of pregnancy [27]. This previous study showed that there was a significantly higher proportion of drinkers among older women after becoming aware of pregnancy compared to younger women. Similarly, the US Centers for Disease Control and Prevention (CDC) reported higher rates of alcohol consumption among pregnant women aged ≥ 35 years compared to pregnant women aged 24–34 years [28]. In addition, according to the National Health and Nutrition Survey of 2019 in Japan, the age-specific proportions of Japanese women who drank ≥ 20 g per day of alcohol, which increased the risk of lifestyle-related diseases, were 5.3% for women in their 20s, 11.7% for women in their 30s, and 13.9% for women in their 40s, among the general population [29]. Thus, age was a risk factor for drinking habits among women on ART treatment, as well as among women in the general population and in pregnancy. 

Women who had not experienced childbirth had a significantly higher prevalence of habitual drinking compared to those who had given birth. This result was similar to that of a previous study reporting that women without a history of childbirth tend to drink and are unable to stop their drinking before they become aware of their pregnancy, compared to those with a history of childbirth [27]. A potential explanation for this is the awareness of parenthood. A previous study demonstrated that parenthood was associated with less frequent drinking compared to pre-pregnancy [30]. In the present study, parenthood through childbirth may contribute to less alcohol consumption among women on ART treatment. Another potential explanation is alcohol education during pregnancy. In Japan, women are more likely to be informed about the risks of alcohol consumption to childbirth during their pregnancy through a Maternal and Child Health Handbook and mother or parent classes provided by municipal governments. According to a previous study in Japan, the knowledge of the health risks of alcohol use to the fetus has been shown to be associated with a reduced risk of alcohol use during pregnancy [31]. Therefore, because women who have already experienced pregnancy and childbirth may have had more opportunities to learn about the risks of alcohol consumption during pregnancy, they may be less likely to drink. 

Among women on ART treatment, working women had a higher prevalence of habitual drinking than those who did not work. This result is consistent with a previous study showing that women who were working consumed significantly more alcohol before becoming aware of their pregnancy compared to women who were not working [27]. Especially in Japan, “NOMIKAI” (drinking with co-workers) is a culture unique to Japanese companies, and in terms of work status, alcohol use is often an important part of social life, indicating that working women have a high opportunity to drink with work colleagues at social events [32,33]. One national survey of alcohol use among Japanese women showed that working women were at higher risk of harmful alcohol use than non-working women [33]. These cultural characteristics in Japan may, therefore, influence the association between employment status and alcohol use among Japanese women undergoing fertility treatment. Furthermore, a previous study showed the socioeconomic interactions of the association between workplace and women’s drinking. Working outside the home is protective against excessive alcohol use among women of low socioeconomic status (SES) but harmful among women of high SES [34]. Considering the current situation of infertility treatment in Japan, the target population of this study is a high SES group; therefore, the aforementioned previous study supports the findings of the present study. 

The partner’s drinking status was significantly associated with habitual drinking among women undergoing ART treatment. This result is similar to that of several previous studies on drinking among pregnant women worldwide [35]. Husbands and wives are said to show similar drinking patterns, the so-called “assortative mating” [30]. A longitudinal study in the USA showed that because husbands and wives have married similar individuals and have shared life experiences that have influenced the couples’ drinking, women’s alcohol consumption after marriage was associated with their partners’ alcohol consumption before marriage [36]. Another study in the Netherlands demonstrated that women were more likely to drink during pregnancy when they thought that their partners agreed with women’s prenatal alcohol use, and women drinking during pregnancy more often had partners whom they perceived and who themselves reported that they did not consider prenatal alcohol abstinence important [35]. Thus, women’s drinking habits may be strongly influenced by their partners’ drinking habits. Furthermore, a previous study in the USA reported significantly lower fertility rates among married couples drinking ≥50 g per week of alcohol during ART treatment compared with couples drinking <50 g per week [11]. Therefore, interventions on drinking habits, not only for women on ART treatment but also for their partners, may be important for a healthy pregnancy.

While previous studies have reported that mental health was associated with alcohol use disorders (e.g., alcohol abuse, alcohol addiction, and alcohol dependence), this study did not find any associations between habitual drinking and psychological distress or insomnia. [37] Although the reasons for this remain unclear, one factor that could have influenced the outcome of this study is the unique definition of habitual drinking adopted. The present study defines habitual drinking as ≥2 *go* (46 g of ethanol) per week, and this amount may not be considered a large amount of alcohol consumption for the general population. Our study suggests that alcohol consumption of ≥2 *go* per week is influenced by social factors such as partner’s drinking status and working status, as well as an assortment of educational factors such as history of childbirth. These social factors surrounding patients are a potential barrier to the effective deployment of educational efforts concerning alcohol consumption for women undergoing ART treatment in Japan.

The strengths of this study are the large sample size and the multivariable analysis, which investigated a wide range of factors, including the patients’ social factors, lifestyle, fertility treatment status, and employment status. On the other hand, our study has the following three limitations: First, the J-FEMA study used a self-administered questionnaire; therefore, the possibility of misclassification due to recall bias cannot be ruled out. Furthermore, non-response bias also cannot be ruled out because the patients who consumed alcohol might have chosen not to answer questions on their drinking status. Second, because of the cross-sectional design of the present study, we could not survey changes in drinking status before and after ART treatment. Further, longitudinal studies are therefore needed to identify the factors associated with changes in drinking habits during ART treatment. Third, the diagnostic data on alcohol use disorders (e.g., alcohol abuse, alcohol addiction, and alcohol dependence) of the patients and their families were not obtained. However, the age-adjusted prevalence of alcohol dependence in women according to the ICD-10 diagnostic criteria (code F10.2) is as small as 0.1% in Japan, and thus, the impact of these diseases on the results of our study should be negligible [38].

To encourage healthy conception, it is important for physicians to advise women on sobriety and on avoiding excessive drinking during ART treatment [39]. The factors associated with drinking habits during ART treatment that were identified in this study may be useful for proactively monitoring the drinking status of women who undergo ART treatment, for example, assessing patients’ drinking status via routine and regular questionnaire-based assessments conducted throughout the duration of fertility treatment. Furthermore, this study demonstrated that social and environmental factors, such as lost opportunities for drinking education due to lack of childbirth history, partner’s drinking status, and employment, may be significant risk factors for drinking habits for women on ART treatment. Therefore, drinking education may be necessary not only for women undergoing ART treatment but also for their partners and workplaces in order to accomplish a healthy pregnancy.

## 5. Conclusions

In our study, 15% of women undergoing ART treatment habitually consumed alcohol, and habitual drinking in this setting was significantly associated with older age, no history of childbirth, partner’s drinking status, and working status. Encouraging drinking education for women on ART treatment, as well as their partners and workplace, may be important for healthy pregnancy and childbirth. Further, longitudinal studies are required to clarify the causal relationship between drinking behavior in women undergoing ART treatment and the factors identified in this study.

## Figures and Tables

**Table 1:** The prevalence of characteristics according to drinking status among 1017 women undergoing ART treatment aged 20 years and over.

Variables Categories	Number (%)						Habitual Drinking Rate (%)	*p* Value **
	Total		Not Habitual Drinking		Habitual Drinking			
N	1017		859	(84.5)	158	(15.5)	15.5	
Age (years)								
<35	197	(19.6)	179	(21.1)	18	(11.6)	9.1	**0.01**
≥35	806	(80.4)	669	(78.9)	137	(88.3)	17.0	
Educational background *								
Higher	528	(52.2)	434	(50.8)	94	(59.9)	17.8	**<0.01**
Lower	483	(47.8)	420	(49.2)	63	(40.1)	13.0	
Partner’s drinking status								
Not drinking	291	(28.8)	280	(32.8)	11	(7.0)	3.8	**<0.01**
Drinking	720	(71.2)	573	(67.2)	147	(93.0)	20.4	
Duration of infertility (year)								
<2	250	(25.2)	213	(25.4)	37	(23.6)	14.8	0.62
≥2	744	(74.8)	624	(74.6)	120	(76.4)	16.1	
IVF cycle								
<6	644	(76.2)	545	(75.8)	99	(78.6)	15.4	0.50
≥6	201	(23.8)	174	(24.2)	27	(21.4)	13.4	
History of childbirth								
Yes	202	(38.4)	179	(41.1)	23	(25.6)	11.4	**0.01**
No	324	(61.6)	257	(58.9)	67	(74.4)	20.7	
K6								
<13	940	(93.8)	791	(93.4)	149	(96.1)	15.9	0.19
≥13	62	(6.2)	56	(6.6)	6	(3.9)	9.7	
ISI-J								
<10	791	(78.7)	664	(78.4)	127	(80.4)	16.1	0.58
≥10	214	(21.3)	183	(21.6)	31	(19.6)	14.5	
Employment type								
Non-worker	273	(27.1)	249	(29.3)	24	(15.3)	8.8	**<0.01**
Worker	735	(72.9)	602	(70.7)	133	(84.7)	18.1	
Company size (employees)								
<50	232	(32.3)	193	(32.8)	39	(29.8)	16.8	0.20
50–999	249	(34.6)	209	(35.5)	40	(30.5)	16.1	
≥1000	238	(33.1)	186	(31.6)	52	(39.7)	21.8	

Notes: N: number of women; IVF: in vitro fertilization; ISI-J: Insomnia Severity Index in Japan. *: The educational background that is university or graduate school as the last education was defined as ‘higher educational background’, and junior high school, high school, junior college, vocational school, and others as the last education as ‘lower educational background’. **: Pearson χ^2^ test. Bold font indicates statistically significant values.

**Table 2:** Crude and multivariable-adjusted odds ratios (ORs) of habitual drinking among women undergoing ART treatment.

Variables Categories	Crude OR (95% CI)	*p* Value ***	Multivariate OR ** (95% CI)	*p* Value ***
Age (years)				
<35	1.00		1.00	
≥35	2.04 (1.21–3.42)	**0.01**	2.27 (0.99–5.21)	0.053
Educational background *				
Higher	1.00		1.00	
Lower	0.69 (0.49–0.98)	**0.04**	0.78 (0.48–1.28)	0.33
Partner’s drinking status				
Not drinking	1.00		1.00	
Drinking	6.53 (3.48–12.25)	**<0.01**	4.26 (1.98–9.16)	**<0.01**
History of childbirth				
Yes	1.00		1.00	
No	2.03 (1.22–3.38)	**0.01**	1.84 (1.08–3.12)	**0.02**
Employment type				
Non-worker	1.00		1.00	
Worker	2.29 (1.45–3.63)	**<0.01**	1.77 (1.00–3.14)	0.050

Notes: OR: Odds ratio; CI: Confidence interval. *: The educational background that is university or graduate school as the last education was defined as ‘higher educational background’, and junior high school, high school, junior college, vocational school, and others as the last education as ‘lower educational background’. **: Each OR was adjusted for all other variables in the table. ***: Bold font indicates statistically significant values.

## Data Availability

The data that support the findings of this study are available on request from the corresponding author. The data are not publicly available due to privacy or ethical restrictions.
